# An Innovative Approach with [^68^Ga]Ga-PSMA PET/CT: The Relationship Between PRIMARY Scores and Clinical and Histopathological Findings

**DOI:** 10.3390/diagnostics15141779

**Published:** 2025-07-15

**Authors:** Gozde Mutevelizade, Bilal Cagri Bozdemir, Nazim Aydin, Elvan Sayit

**Affiliations:** 1 Department of Nuclear Medicine, School of Medicine, Manisa Celal Bayar University, 45030 Manisa, Turkey; bozdemirc@gmail.com (B.C.B.); elvansayit@hotmail.com (E.S.); 2 Department of Nuclear Medicine, Prof. Dr. Cemil Tascıoglu Hospital, University of Health Sciences, 34785 Istanbul, Turkey; nazimaydin94@gmail.com

**Keywords:** PRIMARY score, [^68^Ga]Ga-PSMA PET/CT, prostate cancer

## Abstract

**Background/Objectives**: The aim of this study was to investigate the relationship between the PRIMARY score derived from [^68^Ga]Ga-PSMA PET/CT and key clinical and pathological parameters of prostate cancer aggressiveness, including the PSA level, ISUP Grade Group, and D’Amico risk classification, in patients with biopsy-proven prostate cancer. A secondary aim was to evaluate the interobserver agreement of the PRIMARY score in routine clinical practice. **Methods**: This retrospective analysis included 51 patients with histopathologically confirmed prostate adenocarcinoma who underwent [^68^Ga]Ga-PSMA PET/CT imaging for staging. PRIMARY scores were determined based on the intraprostatic uptake pattern, intensity, and zonal localization. These scores were compared with PSA levels, ISUP GG, D’Amico risk classification, and histopathological features such as the cribriform pattern, intraductal carcinoma, perineural invasion, extraprostatic extension, and lymphovascular invasion. The PRIMARY scores were independently assigned by a total of three nuclear medicine physicians, and interobserver agreement was calculated using Fleiss’ kappa analysis. **Results**: Significant associations were found between the PRIMARY scores and the PSA level, ISUP Grade Group, and D’Amico risk classification. The most prevalent score was PRIMARY 5 (54.9%), which was significantly associated with ISUP GG 5 and the high-risk category in D’Amico classification. Among patients with PRIMARY Score 2, a substantial proportion (64.7%) had ISUP GG ≥ 3, and 58.8% were in the high-risk group, highlighting the limitations of binary PRIMARY classification. No statistically significant correlations were found between the PRIMARY scores and specific histopathologic features. Interobserver agreement was excellent (κ = 0.833). **Conclusions**: The PRIMARY score demonstrates high reproducibility and clinical relevance in stratifying prostate cancer aggressiveness. However, the findings challenge the reliability of binary classifications, particularly for patients with Score 2, who may still harbor high-grade disease. Integrating imaging-based scores with clinical and histopathological data is essential, particularly for accurate staging and decision-making regarding active surveillance.

## 1. Introduction

Prostate cancer (PCa) represents a major global health concern, with its incidence steadily increasing worldwide. According to GLOBOCAN 2022 data, PCa is the second most commonly diagnosed cancer among men globally, accounting for approximately 1.5 million new cases and 397,000 deaths. It ranks as the most frequently diagnosed male cancer in 118 out of 185 countries, reflecting its widespread prevalence, and remains the fifth leading cause of cancer-related mortality among men. Incidence rates are nearly three times higher in transitioned countries than transitioning ones (35.5 vs. 12.6 per 100,000, respectively), likely due to differences in healthcare access, screening programs, and diagnostic infrastructure [1].

Prostate cancer management is guided by histopathological evaluation, serum biomarkers, and imaging techniques. Classifying prostate cancer patients based on their risk status significantly influences treatment management and plays a crucial role in predicting treatment response. The Gleason grading system and D’Amico risk classification are widely used to assess tumor aggressiveness and guide treatment decisions [2,3]. However, despite these established methods, there is a growing interest in refining prognostic models through advanced imaging modalities [4,5]. In parallel to molecular imaging approaches, recent radiomic and machine learning models have been proposed for the non-invasive assessment of tumor aggressiveness. For instance, Telecan et al. applied texture-based machine learning to T2-weighted MRI to predict clinically significant prostate cancer and to differentiate individual ISUP Grade Groups. These results highlight the growing role of data-driven imaging biomarkers in personalized risk stratification [6].

In recent years, Gallium-68 prostate-specific membrane antigen ([^68^Ga]Ga-PSMA) [Glu-NH-CONH-Lys-(Ahx)-[Ga-68(HBED-CC)] positron emission tomography/computed tomography (PET/CT) has become increasingly valuable for staging, restaging, treatment planning, and evaluating treatment response in patients with PCa. Furthermore, [^68^Ga]Ga-PSMA PET/CT has emerged as a promising imaging modality for tumor characterization, enhancing diagnostic accuracy and improving clinical decision-making [7]. Given the increasing use of [^68^Ga]Ga-PSMA PET/CT in clinical practice, understanding its role in predicting histopathological characteristics and risk stratification is of significant interest. [^68^Ga]Ga-PSMA PET/CT improves prostate cancer staging, with intraprostatic PSMA intensity potentially serving as a predictor of clinically relevant oncological outcomes. Evidence indicates that intraprostatic [^68^Ga]Ga-PSMA intensity has prognostic value for adverse pathological features and progression-free survival (PFS), underscoring its potential as a significant biomarker in localized prostate cancer [8].

Imaging plays a pivotal role in the diagnostic pathway of prostate cancer, particularly in identifying candidates for biopsy. Accurate detection and characterization of clinically significant prostate cancer (csPCa) are critical to ensuring appropriate clinical management and avoiding overdiagnosis or overtreatment. Among the available modalities, multiparametric magnetic resonance imaging (mpMRI) has emerged as a valuable tool that improves the detection of csPCa and reduces the number of unnecessary transrectal ultrasound (TRUS)-guided biopsies (bx) by better targeting suspicious lesions [9]. However, mpMRI alone has limitations in sensitivity and interobserver variability. Aiming to overcome these challenges, the PRIMARY trial demonstrated that the combination of PSMA PET/CT and MRI significantly improves sensitivity and negative predictive value in detecting csPCa compared to MRI alone. These findings underscore the growing utility of [^68^Ga]Ga-PSMA PET/CT in enhancing diagnostic accuracy and guiding more selective and effective biopsy strategies [10].

Following the initial results of the PRIMARY trial, a post hoc analysis was conducted in biopsy-naïve populations to improve pre-biopsy detection of csPCa. This analysis led to the development of the PRIMARY score, a five-category classification system based on intraprostatic [^68^Ga]Ga-PSMA PET/CT findings. Integrating tumor localization, uptake patterns (focal vs. diffuse), and PSMA intensity (SUVmax), the PRIMARY score was established to standardize lesion interpretation and enhance the differentiation of csPCa from clinically insignificant disease [11]. Previous studies have mainly evaluated the performance of the PRIMARY scoring system in detecting csPCa among biopsy-naïve patients. In contrast, its relationship with tumor aggressiveness and histopathological characteristics in biopsy-confirmed cases has yet to be adequately investigated. In our setting, national reimbursement policies restrict the use of [^68^Ga]Ga-PSMA PET/CT to patients with a biopsy-confirmed Gleason Score (GS) of ≥7. Consequently, all the patients included in this study had already been diagnosed with csPCa, shifting the objective from detection to the stratification of tumor aggressiveness.

While traditional frameworks such as the EAU and NCCN guidelines rely on biopsy-confirmed criteria (e.g., PSA level, ISUP grade, and clinical stage) for risk stratification, the PRIMARY score, derived from PSMA PET/CT findings, offers a non-invasive, imaging-based alternative. The PRIMARY score has been included in the recent EANM procedural recommendations and builds upon PSMA PET/CT, a modality now endorsed by the EAU 2024 guideline as the preferred staging tool in high-risk prostate cancer [12,13]. Although prior studies have primarily focused on its utility in biopsy-naïve populations to guide initial biopsy decisions, limited data are available on its performance in biopsy-confirmed prostate cancer patients. In clinical practice, many patients undergo PSMA PET/CT following histologic diagnosis, necessitating further evaluation of the score’s applicability in this setting. The aim of this study, therefore, was to assess whether the PRIMARY score remains clinically relevant beyond diagnosis by examining its correlation with tumor aggressiveness markers. Such insights may help expand its utility to staging-based risk stratification and therapeutic decision-making.

Therefore, the primary objective of this study is to investigate the relationship between the PRIMARY score and established indicators of tumor aggressiveness, including ISUP Grade Group (GG), serum prostate-specific antigen (PSA) levels, and histopathological characteristics, in a cohort of patients with biopsy-confirmed csPCa. The aim of this evaluation is to further elucidate the clinical utility of the PRIMARY scoring system in risk stratification, thereby contributing to the refinement of individualized treatment strategies. Moreover, recognizing that the clinical applicability of any imaging-based scoring system depends on its consistency, interreader agreement was assessed to determine the reproducibility of PRIMARY scores across independent observers.

## 2. Materials and Methods

### 2.1. Patient Selection and Study Design

This retrospective study involved evaluating the data of 568 patients with histopathologically proven prostate cancer who underwent [^68^Ga]Ga-PSMA PET/CT imaging for staging at our hospital between July 2021 and May 2024. Patients who had not undergone prostatectomy, chemotherapy, hormone therapy, or radiotherapy before [^68^Ga]Ga-PSMA PET/CT imaging and had complete laboratory and pathological records were included. Patients with missing clinical data, primary tumors indistinguishable from the surrounding prostate tissue, or a history of prostatectomy, chemotherapy, hormone therapy, or radiotherapy before [^68^Ga]Ga-PSMA PET/CT were excluded from the study. From this cohort, 51 patients who met the inclusion criteria and underwent [^68^Ga]Ga-PSMA PET/CT for staging prior to prostatectomy, followed by total or transurethral prostatectomy (TUR-P), were selected for further analysis. All patients had a confirmed diagnosis of prostate adenocarcinoma through 10–12 core TRUS-bx before imaging. Serum PSA levels were measured 2 weeks at most before the [^68^Ga]Ga-PSMA PET/CT scan. For each patient, age, Gleason Score, ISUP GG determined by prostatectomy, D’Amico risk stratification group, serum PSA levels, and histopathological findings (cribriformity, intraductal carcinoma, perineural invasion, extraprostatic spread, lymphovascular invasion) were recorded and analyzed. The study was reviewed and approved by the institutional ethics committee (decree number: 20.478.486/2548). All procedures complied with the ethical standards of the institutional research committee and the Helsinki Declaration and its later amendments. Written informed consent was obtained from all the patients according to our institution’s rules.

### 2.2. Preparation of PSMA-Targeting Ligand and [^68^Ga]Ga-PSMA PET/CT Imaging Protocol

[^68^Ga]Ga-PSMA I&T was synthesized following a fully automated, Good Manufacturing Practice-compliant procedure using a Good Radiopharmaceutical Practice module (ITG iQS-TS) connected to a ^68^Ge/^68^Ga generator (1.11-GBq, ITM) and equipped with a disposable single-use cassette kit. A standardized labeling sequence with 25 µg of unlabeled PSMA I&T was used. The ^68^Ga peptides were synthesized using a cationic purification method, with 25 µg of peptide used for the reaction. The labeling efficiency and radiochemical purity (≥95%) were determined using radio-high-performance liquid chromatography.

Patient preparation, acquisition protocols, and reconstruction parameters were standardized for all patients. Sixty minutes after an intravenous injection of 2.2–2.5 MBq/kg [^68^Ga]Ga-PSMA, the patients were scanned from the vertex to the proximal thigh. Combined (fusion) images of the patients were acquired using a hybrid PET/CT system (GE Discovery IQ, Waukesha, WI, USA). CT acquisition (16-slice; 120 kVp; 90 mA) was immediately followed by multibed PET acquisition (2 min/bed position; 8–10 beds per patient). Patients were allowed to breathe normally. Intravenous contrast material was not used for the PET/CT scan. The maximum intensity projection and attenuation-corrected PET/CT fusion images were evaluated in three orthogonal planes (i.e., axial, coronal, and sagittal).

### 2.3. Image Analysis

Three nuclear medicine physicians from the same institution with substantial experience in [^68^Ga]Ga-PSMA PET/CT interpretation independently evaluated each scan. All readings were performed using the same PET/CT scanner (GE Discovery IQ, Waukesha, WI, USA) and identical workstation software (AW VolumeShare 7 (AW 4.7), version 16.0-8.75 (GE Healthcare, Waukesha, WI, USA)). The readers were blinded to each other’s assessments and all clinical, histopathological, and laboratory data. To ensure consistency in the PRIMARY score interpretation, a standardized training session based on Emmett et al. [11]’s original criteria was held prior to the review process. Scoring was performed at separate time points to minimize potential bias. Any discrepancies between readers were resolved through consensus discussion. These consensus scores were used in all subsequent statistical analyses. Interobserver agreement was quantified using Fleiss’ kappa (κ) analysis.

PRIMARY scores were assigned based on prostate involvement patterns, including the zone of involvement—transition zone (TZ), peripheral zone (PZ), or central zone (CZ)—as well as the uptake pattern (diffuse or focal) and uptake intensity. A descriptive summary of the 5-point PRIMARY scoring system is provided in Table 1 [11]. These scores were then compared with the ISUP Grade Group, serum PSA levels at the time of [^68^Ga]Ga-PSMA PET/CT, and histopathological features, including cribriform pattern, intraductal carcinoma, perineural invasion, extraprostatic extension, and lymphovascular invasion.

### 2.4. Statistical Analysis

The primary aim of the statistical analysis was to investigate whether the PRIMARY score is associated with key clinical and pathological indicators of tumor aggressiveness, including PSA levels, ISUP Grade Group, and D’Amico risk classification. These relationships were examined to evaluate the potential utility of the PRIMARY score in the risk stratification of patients with biopsy-confirmed prostate cancer. The normality of the data distribution was evaluated using the Shapiro–Wilk test, which showed a non-normal distribution. Accordingly, non-parametric methods were used. Descriptive statistics for quantitative variables included means, standard deviations (SDs), medians with interquartile ranges (IQRs), and ranges. Categorical variables were summarized using frequencies and percentages. Fisher’s exact test was used to examine associations between categorical variables, including PRIMARY score categories and clinical/pathological parameters. For comparisons of independent groups, the Kruskal–Wallis test was applied to assess differences in PSA levels across PRIMARY score categories, while the Mann–Whitney U test was used for pairwise comparisons of PSA levels between specific score groups. When statistically significant differences were identified, post hoc pairwise comparisons were conducted using the Mann–Whitney U test with Bonferroni correction. Receiver operating characteristic (ROC) analysis was used to determine the optimal PSA cut-off value for distinguishing between low and high PRIMARY scores. Interobserver agreement among the three nuclear medicine physicians assigning PRIMARY scores was assessed using Fleiss’ kappa (κ), interpreted as follows: ≤0.20 (poor), 0.21–0.40 (fair), 0.41–0.60 (moderate), 0.61–0.80 (substantial), and ≥0.80 (almost perfect agreement). All statistical analyses were performed using IBM SPSS Statistics (version 26.0; IBM Corp., Armonk, NY, USA), and a *p*-value < 0.05 was considered statistically significant.

## 3. Results

### 3.1. Patient Characteristics

**Figure 1:**
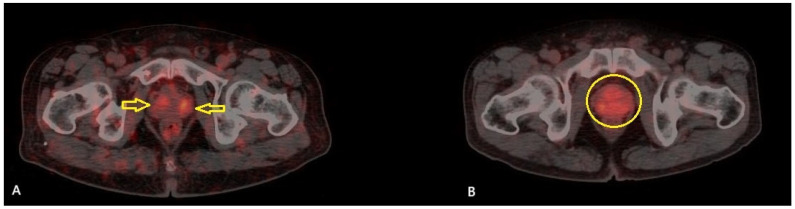
(**A**): Axial [^68^Ga]Ga-PSMA PET/CT fusion image of a 70-year-old male with high-risk prostate cancer (D’Amico classification), ISUP Grade Group 5 (Gleason 5 + 4), and a PSA level of 5.9 ng/mL, demonstrating increased symmetrical PSMA uptake in the transition zone (yellow arrows) (SUVmax: 5.3). (**B**): Axial [^68^Ga]Ga-PSMA PET/CT fusion image of a 65-year-old male with high-risk prostate cancer (D’Amico classification), ISUP Grade Group 5 (Gleason 4 + 5), and a PSA level of 26.7 ng/mL, demonstrating diffuse PSMA uptake in the transition zone (yellow circle) (SUVmax: 4.6). Both lesions received a PRIMARY Score of 2 from all three readers.

A total of 51 patients were included in the study. The mean age of the patients was 71.2 ± 7.4 years (range: 54–89), and the median serum PSA level was 20.3 ng/mL (IQR: 8.45–53.95). Among the patients, TUR-P was performed in 32 (62.7%) patients, while 19 (37.3%) underwent total prostatectomy. According to the pathology results, 11 patients (21.6%) were classified as GG 2, 4 patients (7.8%) as GG 3, 1 patient (2%) as GG 4, and 35 patients (68.6%) as GG 5. The patients were classified according to the D’Amico risk stratification system: 39 patients (76.5%) were classified as high risk, while 12 (23.5%) were in the intermediate-risk group. Based on the PRIMARY score analysis, 17 patients (33.3%) were categorized as PRIMARY Score 2, 6 patients (11.8%) as PRIMARY Score 4, and 28 patients (54.9%) as PRIMARY Score 5. None of the patients in this cohort was classified as PRIMARY Score 1 or 3. A summary of the clinical and histopathological characteristics of the study cohort is presented in Table 2. Among the 17 patients categorized as PRIMARY Score 2, 11 (64.7%) were classified as ISUP GG ≥ 3, and 10 (58.8%) were in the high-risk category according to the D’Amico classification (Figure 1).

### 3.2. Association Between PRIMARY Score and Clinical Parameters

In the Score 5 group, 26 patients (92.9%) were classified as ISUP GG 5, and 22 (78.6%) were assigned to the high-risk category. Statistical analysis demonstrated significant differences between the PRIMARY scores and ISUP GG and D’Amico risk groups (*p* < 0.001). The distribution of ISUP Grade Groups and histopathological features according to PRIMARY score categories is summarized in Table 3.

The relationship between the PRIMARY scores and PSA levels was analyzed using the Kruskal–Wallis test, which yielded a significant result (*p* = 0.046). Bonferroni correction was applied to account for multiple comparisons (adjusted significance threshold: *p* = 0.0167). Post hoc Mann–Whitney U tests were conducted to compare groups, revealing variations in PSA levels among different categories. Table 4 presents the pairwise comparisons of the PSA levels among the PRIMARY score groups, with a statistically significant difference observed only between PRIMARY Scores 4 and 5 (*p* = 0.0160).

### 3.3. Associations with PSA and ISUP Grade

Additionally, the PRIMARY scores were categorized into two groups: Scores 4 and 5 were classified as high-PRIMARY and Score 2 as low-PRIMARY. A ROC analysis was performed to assess whether PSA levels could effectively distinguish between high- and low-PRIMARY groups. However, no significant PSA cut-off value was identified (*p* > 0.05).

The association between the ISUP GG and PSA levels was also assessed using the Kruskal–Wallis test, demonstrating a significant difference (*p* = 0.003). Since there was only one patient with ISUP GG 4, this case was excluded from the statistical analysis to ensure reliable group comparisons. To adjust for multiple comparisons, Bonferroni correction was applied (adjusted significance threshold: *p* = 0.0167). Post hoc Mann–Whitney U tests identified significant differences in PSA levels between GG 2 and GG 3 (*p* = 0.011) as well as between GG 2 and GG 5 (*p* < 0.001) (Table 5).

Patient age was compared across the PRIMARY scores, high-PRIMARY classification, ISUP GG, and D’Amico risk groups; however, no statistically significant differences were observed in any of these comparisons (*p* > 0.05). This result suggests that patient age does not significantly impact tumor grading, risk stratification, or PRIMARY scoring, indicating that age alone may not be a determining factor in these classifications.

### 3.4. Histopathological Correlation and Interobserver Agreement

Two other independent nuclear medicine physicians evaluated the same [^68^Ga]Ga-PSMA PET/CT images, bringing the total number of readers to three, all of whom were blinded to each other’s assessments and patient information. Interobserver agreement was assessed using Fleiss’ kappa analysis, which demonstrated a strong level of concordance among raters in terms of PRIMARY scoring (κ = 0.833; 95% CI: 0.717–0.950; *p* < 0.001). Among the rating categories, the highest agreement was observed for Score 2 (κ = 0.877) and 5 (κ = 0.894).

The relationship between the PRIMARY score and histopathological features was investigated to determine whether PRIMARY scoring is associated with specific pathological characteristics, including cribriform pattern, intraductal carcinoma, perineural invasion, extraprostatic extension, and lymphovascular invasion. However, no statistically significant relationships were found between the histopathological features and PRIMARY scores (*p* > 0.05). The same analysis was also performed using patients in the high-PRIMARY group (PRIMARY Scores 4–5). No statistically significant associations were found between the histopathological features and the high-PRIMARY group, although the *p*-value indicated borderline significance at 0.058.

## 4. Discussion

In this study, we evaluated the clinical relevance of the PRIMARY score, an imaging-based 5-point visual scoring system derived from [^68^Ga]Ga-PSMA PET/CT in patients with biopsy-confirmed prostate cancer. Our findings revealed a statistically significant association between higher PRIMARY scores and established indicators of tumor aggressiveness, including the PSA level, ISUP Grade Group, and D’Amico risk classification. Notably, PRIMARY Score 5 was the most prevalent category in our cohort and was significantly associated with ISUP Grade Group 5 and high-risk classification. These results suggest that the PRIMARY score may offer valuable insight into disease severity and intraprostatic tumor burden at the time of initial staging.

### 4.1. Comparison with Existing Literature

Given its superior sensitivity and specificity compared to conventional imaging techniques, [^68^Ga]Ga-PSMA PET/CT is strongly recommended by the leading clinical guideline as the preferred imaging modality for staging prostate cancer [13,14]. This recommendation is supported by strong clinical evidence, including the findings of the proPSMA study, a prospective, randomized trial demonstrating that [^68^Ga]Ga-PSMA PET/CT provides significantly higher accuracy (92% vs. 65%), greater sensitivity (85% vs. 38%), and lower radiation exposure compared to conventional imaging, ultimately leading to improved staging and clinical decision-making [15]. Beyond its role in staging, [^68^Ga]Ga-PSMA PET/CT enhances the accuracy of tumor detection and correct localization. It guides decisions regarding repeat biopsies in patients with a high clinical suspicion of prostate cancer despite previous negative results, thereby improving diagnostic confidence and refining patient management [12].

In their prospective multicenter PRIMARY study, Emmett et al. evaluated the additive diagnostic value of combining [^68^Ga]Ga-PSMA PET/CT with multiparametric MRI (mpMRI) for detecting csPCa. Integrating both modalities significantly improved the negative predictive value (NPV) and sensitivity, reducing unnecessary biopsies and identifying cases of csPCa that were missed by MRI alone. These findings highlight the potential of PSMA PET/CT as an effective complementary tool in patients with high clinical suspicion but inconclusive mpMRI results [10]. As the significance of [^68^Ga]Ga-PSMA PET/CT in prostate cancer imaging has become increasingly recognized, the need for a standardized and systematic approach to its evaluation has gained prominence. Consequently, various scoring and classification systems have been developed to enhance diagnostic accuracy, optimize clinical decision-making, and improve interobserver consistency.

The PSMA Reporting and Data System (PSMA-RADS) Version 1.0 was introduced by Rowe et al. in 2018 [16], aiming to standardize the evaluation of imaging findings and classify lesions based on their malignancy risk. Addressing the limitations identified in the original framework, PSMA-RADS 2.0 has emerged as an enhanced system designed to improve diagnostic accuracy and broaden clinical utility in interpreting PSMA PET/CT results. Importantly, this updated version offers lesion-specific characterization and introduces an Overall RADS Score (ORS), enabling more precise risk stratification, guiding therapeutic decision-making, and ensuring consistency in lesion assessment [16,17]. While PSMA-RADS 2.0 provides a structured framework for characterizing individual lesions based on their likelihood of malignancy, it does not comprehensively address the overall disease burden or tumor staging. The Prostate Cancer Molecular Imaging Standardized Evaluation (PROMISE) criteria were introduced to fill this gap, offering a molecular imaging-based TNM (miTNM) classification for interpreting PSMA-ligand PET/CT findings. The miTNM system, developed within the PROMISE framework, aligns with the traditional clinicopathologic TNM staging system but is specifically adapted for PSMA PET imaging. Additionally, PROMISE incorporates the miPSMA expression score, which categorizes PSMA uptake relative to reference organs, providing insights into tumor biology and potential responses to PSMA-targeted therapies. As PSMA PET/CT becomes increasingly integrated into clinical workflows, the combination of PSMA-RADS for lesion-based characterization and miTNM for whole-body staging may enhance diagnostic accuracy and clinical decision-making [18].

Emmett et al. proposed a 5-point PRIMARY score that incorporates intraprostatic zone-based localization, uptake patterns, and intensity on [^68^Ga]Ga-PSMA PET/CT, demonstrating high diagnostic accuracy for the detection of csPCa, defined as ISUP GG 2 or higher [11]. PSMA expression is not exclusive to malignancy; benign conditions such as benign prostatic hyperplasia (BPH) and low-grade tumors may also exhibit PSMA uptake, potentially leading to false-positive interpretations. To address this challenge, the PRIMARY score integrates both anatomical localization (PZ, TZ, CZ) and uptake characteristics such as distribution pattern and intensity. Given that the majority of csPCa originates in the PZ, focal uptake in this region is considered highly suspicious and is assigned higher scores, such as PRIMARY Score 4. In contrast, diffuse uptake in the TZ, often associated with benign prostatic changes, is typically assigned lower scores, such as Score 2, unless it is focal and intense, which is assigned Score 3. This structured, zone-based approach enhances diagnostic accuracy by minimizing false positives and improving risk stratification in intraprostatic lesion assessment [19]. While PROMISE and PSMA-RADS focus on systemic disease staging and metastasis detection, the PRIMARY score is limited to localized prostate lesions. Unlike PSMA-RADS, which lacks a prostate-specific scoring mechanism, and PROMISE, which is optimized for whole-body staging rather than lesion-specific risk stratification, the PRIMARY score directly assesses tumor burden within the prostate.

In contrast to the original PRIMARY study and its subsequent post hoc analysis, which were primarily conducted in biopsy-naïve populations to enhance the pre-biopsy detection of csPCa, our study focused on patients with histopathologically confirmed prostate cancer [10,11]. This fundamental difference stems from our current national reimbursement policies, which restrict the use of [^68^Ga]Ga-PSMA PET/CT to patients with a confirmed diagnosis. Consequently, our study population represents a more advanced disease profile, with a higher proportion of ISUP GG 5 (68.6%) and patients classified as high-risk according to the D’Amico criteria (76.5%). This likely accounts for the predominance of PRIMARY Score 5 (54.9%) in our cohort, in contrast to the more evenly distributed PRIMARY scores observed in the original trial and subsequent cohorts [20]. In our cohort, PRIMARY scores were significantly related to PSA levels, ISUP GG, and D’Amico risk stratification (*p* < 0.05). The PRIMARY Score 5 category was the most frequently observed (54.9%) and was significantly associated with adverse risk stratification. Specifically, 26 of the 28 patients (92.9%) with PRIMARY Score 5 were classified as ISUP GG 5, and 22 (78.6%) were categorized as high-risk according to the D’Amico classification. This outcome reinforces the PRIMARY score’s clinical value in identifying patients with high tumor burden and biologically aggressive disease. The statistically significant difference in PSA levels between PRIMARY Scores 4 and 5 (*p* = 0.0160) in our cohort supports the clinical relevance of the semi-quantitative threshold (SUVmax > 12) that defines Score 5. This threshold appears to correlate with biologically aggressive disease. Consistent with this observation, a recent study reported that all patients with SUVmax > 12 had csPCa, and 93% of them were classified as ISUP GG ≥ 3, reinforcing the association between intense intraprostatic PSMA uptake and higher-grade disease [4]. These findings strengthen the association between high PSMA uptake intensity and advanced disease, further supporting the diagnostic and prognostic value of PRIMARY Score 5.

### 4.2. Implications for Risk Stratification and Treatment Planning

Beyond its diagnostic utility, the PRIMARY score may also carry important implications for treatment planning in prostate cancer. According to the current NCCN guidelines, a risk-adapted therapeutic approach is recommended, ranging from active surveillance in very low- and low-risk patients to systemic therapy combined with radiotherapy in high-risk and very high-risk cases [14]. As an imaging-derived score available at the time of initial staging, the PRIMARY score could support clinical decision-making by providing early, non-invasive insight into intraprostatic tumor aggressiveness. Patients with high PRIMARY scores (e.g., 4–5) may be more likely to benefit from intensive or multimodal treatment strategies, whereas those with lower scores may be appropriate candidates for conservative management approaches such as active surveillance or focal therapy. Prospective studies are warranted to validate the role of the PRIMARY score in guiding personalized therapeutic decisions. In this context, it is also important to recognize that the PRIMARY score is not intended to function in isolation. Given the expanding role of multiparametric MRI, molecular markers, and integrated diagnostics in prostate cancer assessment, the PRIMARY score should be interpreted as part of a broader multimodal approach. When combined with other clinical, radiological, and molecular parameters, it may further enhance individualized risk stratification and guide treatment planning.

Several studies have proposed a binary classification of the PRIMARY score, designating Scores 1–2 as negative and Scores 3–5 as positive for detecting csPCa. However, our results challenge the reliability of this classification, particularly in patients with biopsy-proven disease undergoing [^68^Ga]Ga-PSMA PET/CT for staging. In our cohort, of the 17 patients (33.3%) classified as PRIMARY Score 2, 11 had ISUP GG ≥ 3, and 10 were in the high-risk category according to the D’Amico classification, despite being assigned to the “negative” category based on their PRIMARY score. These findings challenge the assumption that Score 2 implies low clinical risk. In the study by Guo et al. (2024) [4], the PRIMARY score demonstrated high sensitivity (90.6%) and negative predictive value (85.8%) for detecting csPCa. However, among the patients classified as negative (Scores 1–2), 21 out of 148 (14.2%) were later confirmed by biopsy to have csPCa, translating to a false-negative rate of 9.4%. Among these, about 19% had high-grade tumors (Gleason Grade Group 3 or higher), which typically require active treatment [4]. Similarly, in the study conducted by Emmett et al., which included 227 patients undergoing PSMA PET/CT and mpMRI before biopsy, the PRIMARY score showed excellent overall diagnostic performance, with a sensitivity and specificity of 96.6% and 93.6%, respectively. Among the 78 patients classified as negative (Score 1–2), 5 (6.4%) were later found to have csPCa. While these findings suggest a more favorable profile of false-negative cases compared to Guo et al.’s cohort, they nonetheless indicate that PRIMARY Scores 1–2 cannot fully exclude csPCa [7]. These data and our results suggest that the binary classification of the PRIMARY score may not always be reliable, particularly in real-world post-diagnostic settings. Treating Scores 1–2 as universally “negative” overlooks the potential presence of csPCa. The persistence of csPCa among patients with Score 2 lesions may reflect intratumoral heterogeneity in PSMA expression or zone-related uptake patterns, emphasizing the need for a nuanced, context-specific interpretation of PRIMARY scoring. Therefore, while a negative PRIMARY score can assist in risk stratification, relying solely on this result to guide biopsy decisions or active surveillance may risk underdiagnosing aggressive disease, particularly in patients with ongoing clinical suspicion.

Such limitations have important clinical implications, particularly for active surveillance strategies that rely heavily on imaging-based risk assessment. Active surveillance (AS) is an established approach for managing selected patients with low-risk prostate cancer, aiming to minimize overtreatment. However, our findings raise concerns about relying solely on imaging-based classifications such as the PRIMARY score to guide AS decisions. Despite being categorized as negative in binary classifications proposed by earlier validation studies, a considerable proportion of patients with PRIMARY Score 2 in our cohort exhibited high-grade or high-risk disease. These observations underscore the need for comprehensive clinical and pathological evaluation when considering AS, rather than depending exclusively on imaging-derived scores. Two recent studies specifically evaluated the utility of PRIMARY scoring in guiding active surveillance decisions. In the study by Uslu et al., 17.6% of patients classified as intermediate- or high-risk were assigned PRIMARY Score 1 or 2 [21]. Similarly, Akçay et al. reported that among 37 patients with PRIMARY Scores 1–2, 19 (51%) were found to have csPCa on final pathology following radical prostatectomy [22]. These data underscore that a non-negligible subset of high-risk patients may still be classified into the “negative” or “low” PRIMARY score category, highlighting the potential for clinical underestimation. This reinforces the importance of integrating clinical and pathological data rather than relying solely on imaging-derived classifications for active surveillance decision-making.

In addition to risk group misclassification, age-based biopsy-sparing strategies have also been explored in recent studies. In a recent prospective study, Li et al. proposed that omission or postponement of prostate biopsy might be considered in men under 65 years of age with PRIMARY Scores 1–2, while suggesting that biopsy should still be pursued in men aged 65 or older when clinical suspicion remains high. This approach is promising; however, data from our study in an older, biopsy-confirmed cohort, with a mean age of 71.2 years, demonstrate that a substantial proportion of patients with PRIMARY Score 2 harbored high-risk disease. These results raise the possibility that our observed rates of discordance may, at least in part, be influenced by the older age distribution of our patient population, highlighting the need for careful patient selection when considering biopsy-sparing strategies based solely on imaging scores [23].

### 4.3. Histopathologic Correlation

To the best of our knowledge, there are currently no published studies specifically comparing detailed histopathologic features, such as cribriform pattern, intraductal carcinoma, perineural invasion, extraprostatic extension, or lymphovascular invasion, with the PRIMARY score based on [^68^Ga]Ga-PSMA PET/CT. In our cohort, no statistically significant associations were found between these histologic subtypes and PRIMARY scores (*p* > 0.05), although a borderline trend was noted in the high-PRIMARY group (Scores 4–5) (*p* = 0.058), suggesting a possible association that may become significant with a larger sample size. These findings indicate that while the PRIMARY score may reflect intraprostatic tumor burden and biological aggressiveness, it does not appear to correlate directly with specific microscopic patterns of adverse pathology. Given the prognostic importance of such features, further studies are warranted to determine whether advanced imaging parameters can non-invasively predict histopathologic complexity.

### 4.4. Reproducibility of PRIMARY Scoring

The reliability of [^68^Ga]Ga-PSMA PET/CT imaging in assessing prostate cancer has been extensively evaluated in recent studies, focusing on interobserver and intraobserver agreement. Interobserver agreement in intraprostatic lesion assessment remains a well-known limitation, particularly with multiparametric MRI. A study examining mpMRI using the PI-RADS v2.1 classification highlighted significant interobserver variability, largely due to interpreter dependency [24]. In a systematic review and meta-analysis, Chavoshi et al. reported a pooled interobserver agreement of κ = 0.67 for intraprostatic lesion evaluation based on [^68^Ga]Ga-PSMA PET/CT-MR, indicating substantial yet variable consistency. This emphasizes the need for structured evaluation systems to enhance reproducibility in local tumor assessment [25]. Structured scoring tools, such as the PRIMARY score, have shown promise in overcoming these limitations. A key strength of the PRIMARY scoring system lies in its reproducibility. Emmett et al. demonstrated that PRIMARY scoring achieved higher interobserver agreement than PI-RADS (κ = 0.65 vs. κ = 0.48), despite only a brief, one-hour training session for the readers [7]. In the study by Soyluoğlu et al., interobserver agreement for intraprostatic lesion detection varied with reader experience (κ = 0.74 vs. κ = 0.62), underscoring the influence of expertise on diagnostic consistency [26]. In our study, three independent nuclear medicine physicians had high interobserver agreement, with a Fleiss’ kappa value of 0.833 (95% CI: 0.717–0.950, *p* < 0.001). Notably, the highest agreement was achieved in Score 2 (κ = 0.877) and Score 5 (κ = 0.894). This high level of concordance reinforces the potential utility of PRIMARY scoring as a standardized tool for guiding prostate cancer management and supports its reliability and consistency in routine practice.

### 4.5. Study Limitations

This study has several limitations. First, its retrospective and single-center design may limit the generalizability of the findings. Second, the relatively small sample size limits the statistical power, although the results provide valuable preliminary insights. Third, the cross-sectional nature of the study precludes the evaluation of long-term oncologic outcomes, as no follow-up data were available to correlate the PRIMARY scores with biochemical recurrence or survival. Although biochemical progression is frequently used as a surrogate endpoint in prostate cancer research, it does not fully capture the complexity of long-term disease progression. These factors underscore the need for prospective, multicenter studies with extended follow-up to validate the prognostic utility of the PRIMARY score.

## 5. Conclusions

This study reinforces the clinical utility of the PRIMARY scoring system as a non-invasive imaging tool in patients with biopsy-confirmed prostate cancer. Significant associations were observed between the PRIMARY scores and key clinical indicators, including ISUP Grade Group, PSA levels, and D’Amico risk classification, supporting its role in risk-adapted assessment. The high interobserver agreement further confirms its reproducibility in routine nuclear medicine practice. Importantly, our findings challenge the reliability of binary classifications, particularly for PRIMARY Score 2, which frequently corresponded to high-grade disease. These results underscore the need for cautious interpretation and support the integration of PRIMARY scores with other diagnostic tools. Future studies combining the PRIMARY score with molecular biomarkers, genomic classifiers, and histopathologic features may enhance individualized treatment planning and inform the selection of active surveillance or targeted therapies.

## Figures and Tables

**Table 1:** Definitions of the 5-point PRIMARY score system used in [^68^Ga]Ga-PSMA PET/CT.

PRIMARY Score	Zone and Pattern	Description
**Score 1**	-	No pattern. Low-grade activity only.
**Score 2**	TZ (Pattern A)	Diffuse TZ uptake, sparing the peripheral margin; may show moderate variation in intensity.
**Score 2**	CZ (Pattern B)	Frequently symmetrical activity; considered Score 4 if it extends to the peripheral margin.
**Score 3**	TZ (Pattern C)	Focal activity is at least twice the background TZ uptake.
**Score 4**	PZ (Pattern D)	Any focal uptake involving the peripheral margin or apex, regardless of intensity.
**Score 5**	-	Intense uptake with SUVmax > 12.

**PZ**: peripheral zone; **TZ**: transition zone; **CZ**: central zone; **SUV**: standardized uptake value.

**Table 2:** Summary of clinical and histopathological characteristics of the study cohort.

Variable	Value (*n* = 51)
Number of patients	51
Mean age (years)	71.2 ± 7.4
PSA level, median (IQR)	20.3 ng/mL (IQR: 8.45–53.95)
TUR-P performed	32 (62.7%)
Total prostatectomy	19 (37.3%)
ISUP Grade Group 2	11 (21.6%)
ISUP Grade Group 3	4 (7.8%)
ISUP Grade Group 4	1 (2.0%)
ISUP Grade Group 5	35 (68.6%)
D’Amico high-risk	39 (76.5%)
D’Amico intermediate-risk	12 (23.5%)
PRIMARY Score 2	17 (33.3%)
PRIMARY Score 4	6 (11.8%)
PRIMARY Score 5	28 (54.9%)
Cribriform pattern	29 (56.9%)
Intraductal carcinoma	11 (21.6%)
Perineural invasion	13 (25.5%)
Extraprostatic extension	12 (23.5%)
Lymphovascular invasion	5 (9.8%)

**PSA**: prostate-specific antigen; **TUR-P***:* transurethral prostatectomy; **ISUP***:* International Society of Urological Pathology.

**Table 3:** Distribution of ISUP Grade Groups and histopathological features according to PRIMARY score categories.

PRIMARY Score	Number of Patients	ISUP GG2	≥ISUP GG3	Cribriformity (+)	Intraductal Ca (+)	PNI (+)	EPE (+)	LVI (+)
2	17	6 (35.3%)	11 (64.7%)	4 (23.5%)	4 (23.5%)	4 (23.5%)	2 (11.8%)	1 (5.9%)
4	6	4 (66.7%)	2 (33.3%)	4 (66.7%)	1 (16.7%)	1 (16.7%)	2 (33.3%)	0 (0.0%)
5	28	1 (3.6%)	27(96.4%)	21 (75%)	6 (21.4%)	8 (28.6%)	8 (28.6%)	4 (14.3%)

**ISUP**: International Society of Urological Pathology; GG: Grade Group; **PNI**: perineural invasion; **EPE**: extraprostatic extension; **LVI**: lymphovascular invasion.

**Table 4:** Comparison of PSA levels among PRIMARY score groups.

Comparison	*p*-Value	Interpretation
PRIMARY Score 2 vs. Score 4	0.234	NS (*p* > 0.0167)
PRIMARY Score 2 vs. Score 5	0.182	NS (*p* > 0.0167)
PRIMARY Score 4 vs. Score 5	**0.0160**	***p*** ** < 0.0167**

**NS:** Non-significant (not statistically significant, *p* > 0.0167 after Bonferroni correction).

**Table 5:** Comparison of PSA levels among ISUP Grade Groups.

Comparison	*p*-Value	Interpretation
ISUP GG2 vs. ISUP GG3	**0.011**	***p*** ** < 0.0167**
ISUP GG2 vs. ISUP GG5	**<0.001**	***p*** ** < 0.0167**
ISUP GG3 vs. ISUP GG5	0.457	NS (*p* > 0.0167)

**NS:** Non-significant (not statistically significant, *p* > 0.0167 after Bonferroni correction).

## Data Availability

The data that support the findings of this study are not publicly available due to ethical and institutional restrictions but are available from the corresponding author upon reasonable request.

## References

[1] Bray F., Laversanne M., Sung H., Ferlay J., Siegel R.L., Soerjomataram I., Jemal A. (2024). Global cancer statistics 2022: GLOBOCAN estimates of incidence and mortality worldwide for 36 cancers in 185 countries. CA Cancer J. Clin..

[2] D’AMico A.V., Whittington R., Malkowicz S.B., Schultz D., Blank K., Broderick G.A., Tomaszewski J.E., Renshaw A.A., Kaplan I., Beard C.J. (1998). Biochemical outcome after radical prostatectomy, external beam radiation therapy, or interstitial radiation therapy for clinically localized prostate cancer. JAMA.

[3] Epstein J.I., Egevad L., Amin M.B., Delahunt B., Srigley J.R., Humphrey P.A., Grading Committee (2016). The 2014 International Society of Urological Pathology (ISUP) Consensus Conference on Gleason Grading of Prostatic Carcinoma: Definition of Grading Patterns and Proposal for a New Grading System. Am. J. Surg. Pathol..

[4] Guo S., Kang F., Ma S., Jiao J., Ren J., Wang J., Zhang J., Qin W. (2024). The PRIMARY Score: Diagnostic Performance and Added Value Compared with MRI in Detecting Clinically Significant Prostate Cancer. Clin. Nucl. Med..

[5] Guglielmo P., Buffi N., Porreca A., Setti L., Aricò D., Muraglia L., Evangelista L. (2025). Current insights on PSMA PET/CT in intermediate-risk prostate cancer: A literature review. Ann. Nucl. Med..

[6] Telecan T., Caraiani C., Boca B., Sipos-Lascu R., Diosan L., Balint Z., Hendea R.M., Andras I., Crisan N., Lupsor-Platon M. (2025). Automatic Characterization of Prostate Suspect Lesions on T2-Weighted Image Acquisitions Using Texture Features and Machine-Learning Methods: A Pilot Study. Diagnostics.

[7] Emmett L., Papa N., Counter W., Calais J., Barbato F., Burger I., Eiber M., Roberts M.J., Agrawal S., Franklin A. (2024). Reproducibility and Accuracy of the PRIMARY Score on PSMA PET and of PI-RADS on Multiparametric MRI for Prostate Cancer Diagnosis Within a Real-World Database. J. Nucl. Med..

[8] Roberts M.J., Morton A., Donato P., Kyle S., Pattison D.A., Thomas P., Coughlin G., Esler R., Dunglison N., Gardiner R.A. (2021). ^68^Ga-PSMA PET/CT tumour intensity pre-operatively predicts adverse pathological outcomes and progression-free survival in localised prostate cancer. Eur. J. Nucl. Med. Mol. Imaging.

[9] Ahmed H.U., El-Shater Bosaily A., Brown L.C., Gabe R., Kaplan R., Parmar M.K., Collaco-Moraes Y., Ward K., Hindley R.G., Freeman A. (2017). PROMIS study group. Diagnostic accuracy of multi-parametric MRI and TRUS biopsy in prostate cancer (PROMIS): A paired validating confirmatory study. Lancet.

[10] Emmett L., Buteau J., Papa N., Moon D., Thompson J., Roberts M.J., Rasiah K., Pattison D.A., Yaxley J., Thomas P. (2021). The Additive Diagnostic Value of Prostate-specific Membrane Antigen Positron Emission Tomography Computed Tomography to Multiparametric Magnetic Resonance Imaging Triage in the Diagnosis of Prostate Cancer (PRIMARY): A Prospective Multicentre Study. Eur. Urol..

[11] Emmett L.M., Papa N., Buteau J., Ho B., Liu V., Roberts M., Thompson J., Moon D., Sheehan-Dare G., Alghazo O. (2022). The PRIMARY Score: Using Intraprostatic 68Ga-PSMA PET/CT Patterns to Optimize Prostate Cancer Diagnosis. J. Nucl. Med..

[12] Fendler W.P., Eiber M., Beheshti M., Bomanji J., Calais J., Ceci F., Cho S.Y., Fanti S., Giesel F.L., Goffin K. (2023). PSMA PET/CT: Joint EANM procedure guideline/SNMMI procedure standard for prostate cancer imaging 2.0. Eur. J. Nucl. Med. Mol. Imaging.

[13] Cornford P., van den Bergh R.C.N., Briers E., Van den Broeck T., Brunckhorst O., Darraugh J., Eberli D., De Meerleer G., De Santis M., Farolfi A. (2024). EAU-EANM-ESTRO-ESUR-ISUP-SIOG Guidelines on Prostate Cancer-2024 Update. Part I: Screening, Diagnosis, and Local Treatment with Curative Intent. Eur. Urol..

[14] Schaeffer E.M., Srinivas S., Adra N., An Y., Bitting R., Chapin B., Cheng H.H., D’Amico A.V., Desai N., Dorff T. (2024). NCCN Guidelines^®^ Insights: Prostate Cancer, Version 3.2024. J. Natl. Compr. Canc. Netw..

[15] Hofman M.S., Lawrentschuk N., Francis R.J., Tang C., Vela I., Thomas P., Rutherford N., Martin J.M., Frydenberg M., Shakher R. (2020). proPSMA Study Group Collaborators. Prostate-specific membrane antigen PET-CT in patients with high-risk prostate cancer before curative-intent surgery or radiotherapy (proPSMA): A prospective, randomised, multicentre study. Lancet.

[16] Rowe S.P., Pienta K.J., Pomper M.G., Gorin M.A. (2018). Proposal for a Structured Reporting System for Prostate-Specific Membrane Antigen-Targeted PET Imaging: PSMA-RADS Version 1.0. J. Nucl. Med..

[17] Werner R.A., Hartrampf P.E., Fendler W.P., Serfling S.E., Derlin T., Higuchi T., Pienta K.J., Gafita A., Hope T.A., Pomper M.G. (2023). Prostate-specific Membrane Antigen Reporting and Data System Version 2.0. Eur. Urol..

[18] Eiber M., Herrmann K., Calais J., Hadaschik B., Giesel F.L., Hartenbach M., Hope T., Reiter R., Maurer T., Weber W.A. (2018). Prostate Cancer Molecular Imaging Standardized Evaluation (PROMISE): Proposed miTNM Classification for the Interpretation of PSMA-Ligand PET/CT. J. Nucl. Med..

[19] Sridhar S., Abouelfetouh Z., Codreanu I., Gupta N., Zhang S., Efstathiou E., Karolyi D.K., Shen S.S., LaViolette P.S., Miles B. (2025). The Role of Dynamic Contrast Enhanced Magnetic Resonance Imaging in Evaluating Prostate Adenocarcinoma: A Partially-Blinded Retrospective Study of a Prostatectomy Patient Cohort With Whole Gland Histopathology Correlation and Application of PI-RADS or TNM Staging. Prostate.

[20] Guo S., Ren J., Meng Q., Zhang B., Jiao J., Han D., Wu P., Ma S., Zhang J., Xing N. (2025). The impact of integrating PRIMARY score or SUVmax with MRI-based risk models for the detection of clinically significant prostate cancer. Eur. J. Nucl. Med. Mol. Imaging.

[21] Uslu H., Şahin D., İbIşoğlu E., Tatoğlu M.T. (2025). PRIMARY scoring in 68Ga-PSMA PET/CT: Correlation with prostate cancer risk groups and its potential impact on active surveillance. Ann. Nucl. Med..

[22] Akcay K., Kibar A., Sahin O.E., Demirbilek M., Beydagi G., Asa S., Aghazada F., Toklu T., Selcuk N.A., Onal B. (2024). Prediction of clinically significant prostate cancer by [^68^Ga]Ga-PSMA-11 PET/CT: A potential tool for selecting patients for active surveillance. Eur. J. Nucl. Med. Mol. Imaging.

[23] Li Y., Yang J., Xiao L., Zhou M., Li J., Cai Y., Gao X., Rominger A., Shi K., Seifert R. (2025). Which patients with negative PSMA-PET imaging can safely avoid biopsy for prostate cancer? a novel step towards PSMA-based biopsy-free strategy. Eur. J. Nucl. Med. Mol. Imaging.

[24] Jóźwiak R., Sobecki P., Lorenc T. (2023). Intraobserver and Interobserver Agreement between Six Radiologists Describing mpMRI Features of Prostate Cancer Using a PI-RADS 2.1 Structured Reporting Scheme. Life.

[25] Chavoshi M., Mirshahvalad S.A., Metser U., Veit-Haibach P. (2022). ^68^Ga-PSMA PET in prostate cancer: A systematic review and meta-analysis of the observer agreement. Eur. J. Nucl. Med. Mol. Imaging.

[26] Soyluoglu S., Korkmaz U., Ozdemir B., Ustun F., Durmus-Altun G. (2021). 68Ga-PSMA-I&T-PET/CT interobserver and intraobserver agreement for prostate cancer: A lesion based and subregional comparison study among observers with different levels of experience. Nucl. Med. Commun..

